# A Protective Role of FAM13A in Human Airway Epithelial Cells Upon Exposure to Cigarette Smoke Extract

**DOI:** 10.3389/fphys.2021.690936

**Published:** 2021-06-07

**Authors:** Qing Chen, Maaike de Vries, Kingsley Okechukwu Nwozor, Jacobien A. Noordhoek, Corry-Anke Brandsma, H. Marike Boezen, Irene H. Heijink

**Affiliations:** ^1^Department of Pathology and Medical Biology, University Medical Center Groningen, University of Groningen, Groningen, Netherlands; ^2^Groningen Research Institute for Asthma and COPD (GRIAC), University Medical Center Groningen, University of Groningen, Groningen, Netherlands; ^3^Department of Epidemiology, University Medical Center Groningen, University of Groningen, Groningen, Netherlands; ^4^Department of Pulmonology, University Medical Center Groningen, University of Groningen, Groningen, Netherlands

**Keywords:** COPD-Chronic obstructive pulmonary disease, FAM13A, airway epithelium, barrier function, CXCL8

## Abstract

**Background:**

Chronic Obstructive Pulmonary Disease (COPD) is a progressive lung disease characterized by chronic inflammation upon inhalation of noxious particles, e.g., cigarette smoke. FAM13A is one of the genes often found to be associated with COPD, however its function in the pathophysiology of COPD is incompletely understood. We studied its role in airway epithelial barrier integrity and cigarette smoke-induced epithelial responses.

**Materials and Methods:**

Protein level and localization of FAM13A was assessed with immunohistochemistry in lung tissue from COPD patients and non-COPD controls. *In vitro*, FAM13A expression was determined in the absence or presence of cigarette smoke extract (CSE) in primary airway epithelial cells (AECs) from COPD patients and controls by western blotting. FAM13A was overexpressed in cell line 16HBE14o- and its effect on barrier function was monitored real-time by electrical resistance. Expression of junctional protein E-cadherin and β-catenin was assessed by western blotting. The secretion of neutrophil attractant CXCL8 upon CSE exposure was measured by ELISA.

**Results:**

FAM13A was strongly expressed in airway epithelium, but significantly weaker in airways of COPD patients compared to non-COPD controls. In COPD-derived AECs, but not those of controls, FAM13A was significantly downregulated by CSE. 16HBE14o- cells overexpressing FAM13A built up epithelial resistance significantly more rapidly, which was accompanied by higher E-cadherin expression and reduced CSE-induced CXCL8 levels.

**Conclusion:**

Our data indicate that the expression of FAM13A is lower in airway epithelium of COPD patients compared to non-COPD controls. In addition, cigarette smoking selectively downregulates airway epithelial expression of FAM13A in COPD patients. This may have important consequences for the pathophysiology of COPD, as the more rapid build-up of epithelial resistance upon FAM13A overexpression suggests improved (re)constitution of barrier function. The reduced epithelial secretion of CXCL8 upon CSE-induced damage suggests that lower FAM13A expression upon cigarette smoking may facilitate epithelial-driven neutrophilia.

## Introduction

Chronic Obstructive Pulmonary Disease (COPD) is a severe and debilitating inflammatory lung disease characterized by persistent airflow limitation and reduced gas exchange. Patients with COPD suffer from severe respiratory symptoms such as chronic cough and shortness of breath, which negatively influence their quality of life (Barnes et al., 2003). It is estimated that 384 million people suffered from COPD in 2010 and COPD is expected to become the third leading cause of death (Adeloye et al., 2015). Important features of COPD are neutrophilic inflammation in the lungs, mucus hypersecretion in the large airways, and abnormal tissue damage and repair responses resulting in (small) airway remodeling and destruction of alveolar tissue.

One of the main risk factors for the development of COPD is exposure to cigarette smoke, which first encounters the airway epithelial cells (AECs) (van der Vaart et al., 2005; Abrams et al., 2014). AECs maintain barrier integrity by forming intercellular contacts, including adherens junctions and tight junctions, to defend the underlying cells from inhaled insults (Hellings and Steelant, 2020). Cigarette smoke causes mitochondrial damage and oxidative stress (Aghapour et al., 2020), leading to epithelial damage, affecting epithelial intercellular contacts (Aghapour et al., 2018). *In vitro*, cigarette smoke extract (CSE) disrupts epithelial junctions, of which E-cadherin is a key constituent (Heijink et al., 2012), being linked to the cytoskeleton by β-catenin. In COPD, the airway epithelium may be less able to recover from this type of damage, as persistent disruption of airway epithelial cell-cell contacts has been observed in COPD (Oldenburger et al., 2014) together with the inability to form cell-cell contacts, polarize and (re)differentiate *in vitro* (Heijink et al., 2014). Defective epithelial barrier function not only leads to increased permeability to inhaled particles (Gangl et al., 2009; Heijink et al., 2012) it also promotes pro-inflammatory activity of the epithelium (Aghapour et al., 2018). AECs, especially when damaged, secrete pro-inflammatory mediators such as CXCL8, which recruits neutrophils (Higham et al., 2018). Repeated injury of the airway epithelium by inhaled particles, such as cigarette smoke, may result in chronic inflammation in susceptible individuals and ultimately lead to chronic bronchitis and/or emphysema in COPD (Barnes et al., 2003; Barnes, 2008; Brusselle et al., 2011).

Although the pathogenesis of COPD is still not fully understood, it is known that its development is influenced by both genetic and environmental factors. Why the airway epithelium of certain individuals is more susceptible to abnormal pro-inflammatory, damage, and repair responses to cigarette smoke is currently unknown. To unravel this individual susceptibility, large scale genome-wide association studies (GWAS) on COPD have been performed, resulting in the identification of multiple susceptibility genes for COPD (Hobbs et al., 2017; van der Plaat et al., 2017). Of interest, these susceptibility genes are often expressed in the airway epithelium. One of such a susceptibility gene is Family with Sequence Similarity 13 A (FAM13A) (Jiang et al., 2016), which was shown to be involved in emphysema susceptibility, and to regulate mitochondrial function (Jiang et al., 2016, 2017; Hawkins and Mora, 2017), and β-catenin stability (Meng and Takeichi, 2009; Jiang et al., 2016). Jiang and co-workers also showed that FAM13A is expressed in lung tissue (Jiang et al., 2016), however they did not study its localization within the tissue. Moreover, it is not understood how altered expression or function contributes to the development of COPD. We hypothesized that FAM13A affects the susceptibility to cigarette smoke in airway epithelial cells. Therefore, in this study we investigated the expression of FAM13A in lung tissue, the effect of cigarette smoke exposure on airway epithelial FAM13A expression and its functional role in airway epithelial barrier function and pro-inflammatory responses in human AECs.

## Materials and Methods

### Subjects and Design of the Study

Human lung tissue for immunohistochemical staining was derived from 11 GOLD stage IV COPD patients, and 9 non-COPD controls (see Table 1 for subject characteristics). Primary AECs were isolated and cultured from tracheobronchial tissue of 9 GOLD stage III and IV COPD patients undergoing lung transplantation (see Table 2 for subject characteristics). The study protocol was consistent with the Research Code of the University Medical Center Groningen^1^ and national ethical and professional guidelines (‘‘Code of conduct; Dutch federation of biomedical scientific societies,’’^2^). Control-derived AECs were obtained from the leftover tracheobronchial tissue of 10 transplantation donor lungs without COPD from whom no information was available. Airway epithelial cultures were obtained by enzymatic treatment and stored in liquid nitrogen before experimentation as described previously (Heijink et al., 2016).

**TABLE 1:** Characteristics of the subjects from whom lung tissue was obtained.

	**Control**	**COPD IV**
Subjects, N	9	12
Female, N	6	8
Age, years (range)	55 (43–62)	52 (47–55)
Smoking status	Ex-smoker	Ex-smoker
FEV1 % predicted	104 (85–127)	17.4 (12–29.9)

*Data are presented as median (range) unless otherwise stated. FEV1: forced expiratory volume in 1 s, the best one among multiple measurements is shown. COPD patients were included based on the Global Initiative for Chronic Obstructive Lung Disease standard.*

**TABLE 2:** Characteristics of COPD patients from whom airway epithelial cells (AECs) were obtained.

**Age**	**Gender**	**Smoking status**	**Packs/year**	**FEV1 (%predicted)**	**FEV1/FVC**	**COPD Stage**
60	F	EX	38	18	18	IV
54	F	EX	35	25	28	IV
56	M	EX	30	31	29	III
63	F	EX	40	22	32	IV
63	M	NS	0	41	45	III
63	M	EX	45	6	40	IV
51	M	EX	30	16	32	IV

*M, male; F, female; EX, ex-smoker; NS, never smoker; FEV1, forced expiratory volume in 1 s; FVC, forced vital capacity.*

### Immunohistochemistry Staining

Localization and expression of FAM13A protein was determined using immunohistochemical staining in lung tissue. Lung tissue was embedded in paraffin and cut into three-μm thick lung sections. Antigen retrieval was done with 10 mM citrate buffer at pH 6.0 followed by avidin/biotin blocking using the Avidin/Biotin blocking kit (Vector Laboratories, Inc., Burlingame, CA 94010). Specific primary antibody against FAM13A (55401-1-AP, Proteintech, Manchester, United Kingdom), E-cadherin (610182, BD Bioscience, Breda, NL), secondary antibody Goat Anti-Rabbit Immunoglobulins/HRP (Dako P0488, Amsterdam, NL), Rabbit Anti-Mouse Immunoglobulins/HRP (Dako P0260, Amsterdam, NL) and tertiary antibody Streptavidin/HRP (Dako P0397, Canada) were used. Positive staining was visualized using VECTOR^®^ NovaRED^TM^ (SK-4800, Vector Laboratories, Canada). The slides were scanned with the Hamamatsu NanoZoomer 2.0HT digital slide scanner, and representative pictures were taken using the Hamamatsu NDP view 2 software.

### Analysis of Immunohistochemistry Staining

The epithelial layer was extracted from airways using Adobe Photoshop (Adobe Inc. California, United States) and ImageJ (Bethesda, Maryland, United States^3^) was used to quantify the intensity and distribution of the staining (Schindelin et al., 2012). NovaRed staining in the images was measured by isolating the signal with color deconvolution plugin by Gabriel Landini (Ruifrok and Johnston, 2001). Thresholding was then used to select the positive pixels, presenting the data as the average intensity of the NovaRed stain of all airways per donor.

### Epithelial Cell Culture

COPD and control-derived primary AECs were derived from fresh bronchial tissue using enzymatic treatment, stored in liquid nitrogen and cultured as described previously (Heijink et al., 2016). In short, cells were seeded into T25 culture flasks coated with 30 μg/ml collagen, 30 μg/ml fibronectin and 10 μg/ml BSA in hormonally supplemented bronchial epithelium growth medium (BEGM; Lonza) with 100 U/ml Penicillin and 100 mg/ml streptomycin. When 90% confluent, the cells were expanded and seeded in duplicates for experiments at passage 3.

The human bronchial epithelial cell line 16HBE14o- was generously offered by D.C. Gruenert (University of California, San Francisco, CA, United States) and the cells were cultured as described previously (Heijink et al., 2007). Briefly, cells were cultured in T25 flasks (Corning, NY, United States) or 24-wells plates coated with 30 μg/ml collagen and 10 μg/ml bovine serum albumin (BSA) in EMEM (Biowhittaker, Verviers, Belgium) with 2 mM GlutaMax (Life Technologies, Paisley, United Kingdom), 100 U/ml Penicillin and 100 mg/ml streptomycin, and 10% fetal calf serum (FCS; Biowhittaker, Verviers, Belgium). When 90% confluent, the cells were passaged or seeded in duplicates into coated 24-wells or 12-wells plates for experiments.

### Cigarette Smoke Extract Preparation

Kentucky 3R4F research-reference cigarettes (The Tobacco Research Institute, University of Kentucky, Lexington, KY, United States) were used to prepare CSE. Filters were removed and the cigarettes were smoked using a hose pump (Watson Marlow 603S, Cornwall, United Kingdom) at a rate of 7 L/h (Pouwels et al., 2016). The smoke of two cigarettes was bubbled through 25 mL EMEM medium and defined as 100% CSE. The extract was used freshly within 15 min after preparation.

### Treatment of the Cells

For CSE exposure, cells were seeded into 24-wells plates at a density of 5 × 10^4^ cells/well, grown for 2–3 days until 90% confluence was reached, and hormonally-deprived (AECs) overnight. Next, cells were stimulated with 0, 10 or 20% CSE or EMEM/BEBM alone as control, for 6 or 24 h before harvesting for RNA isolation, cell lysate preparation and collection of the supernatant.

For FAM13A functional studies, a FAM13A overexpression plasmid was created with pcDNA3.1 backbones and FAM13A clones (NM_001265578.1) by GenScript (Nanjing, China) and plasmid pCMV6-6XL was used as empty vector control. 16HBE14o- cells were seeded in 24−wells plates at a density of 1 × 10^5^ cells/well. The second day, the cells were transfected with 250 ng/well plasmids using 1 μl/well Lipofectamine 2000 transfection reagent in 500 μl OPTIMEM (Life Technologies). After 4 h, the transfection solution was replaced by culture medium. Cells were serum deprived overnight the second day after transfection and treated with CSE as described above.

### Total RNA Extraction and Real-Time PCR

Total RNA of the cells was harvested using TriZol solution (MRC, Cincinnati, United States), RNA was isolated using chloroform and isopropanol, and RNA concentration was measured using a Nanodrop-1000 (ND 2.0; NanoDrop Technologies, Wilmington, DE, United States). One microgram of total RNA from each sample was reverse-transcribed into cDNA using the iScript cDNA synthesis kit (BIO-RAD) according to the manufacturer’s protocol.

qPCR was performed in duplicates with GoTaq(R) Probe qPCR Master Mix kits (Promega Benelux, Leiden) using QuantStudio (Thermo Fisher Scientific, Waltham, United States). B2M and PPIA were used to FAM13A correct for all Taqman probes. FAM13A (Hs00208453_m1), *B2M* (Hs99999907_m1), and *PPIA* (Hs99999904_m1) were purchased from Thermo Fisher Scientific. Data were expressed as 2^–ΔCt^ relative to the appropriate control.

### Western Blotting

Total cell lysates were prepared as described previously (Heijink et al., 2007). Immunodetection was performed using anti−FAM13A (1:1,000; 55401-1-AP, Proteintech, Manchester, United Kingdom), anti-β-catenin (1:1,000; #9587, CST, Leiden, Netherlands), Purified Mouse Anti-E-Cadherin (1:1,000; 610182, BD Transduction Laboratories^TM^, United Stated), anti−β-actin (1:1,000; Santa Cruz Biotechnology, Heidelberg, Germany), polyclonal goat anti-rabbit and rabbit anti-mouse immunoglobulins/HRP (1:2,000; Dako Denmark, Flostrup, Denmark). The blots were developed using SuperSignal^TM^ West Pico PLUS Chemiluminescent Substrate according to the manufacturer’s guidelines (Thermo Fisher Scientific). Quantification of the western blot was performed using software Image Lab (Bio-Rad Laboratories, California, United States).

### ELISA

Supernatant was centrifuged at 500 g for 5 min to remove the cells debris, and aliquoted and stored at −80 degrees. CXCL8 levels were measured in the stored cell-free supernatant using a Duoset ELISA Development Kit (DY208, R and D Systems, Minneapolis, United States), according to the manufacturer’s instructions.

### Electric Cell-Substrate Impedance Sensing (ECIS)

ECIS^®^ instrumentation (Applied Biophysics, Troy, NY, United States) was used to determine the effect of FAM13A on the formation of cell-cell contacts. In brief, 16HBE14o- cells were seeded in 12-wells plates at a density of 2 × 10^5^ cells/well and transfected with 500 ng/well FAM13A plasmids as described above. ECIS arrays (8W10E, Applied Biophysics) were stabilized and coated with collagen and BSA overnight at 37°C, 5% CO_2_. The second day, cells were trypsinized and re-seeded into ECIS arrays at a density of 75,000 cells/array well. We measured low-frequency resistance at 400 Hz as the most sensitive parameter for cell-cell contacts and high-frequency capacitance at 32,000 Hz as the most sensitive parameter for cell-substrate contacts and monitored cells for 3 days (Wegener et al., 2000; Heijink et al., 2010a).

### Statistics

To test for differences between the conditions within groups, we applied the Wilcoxon signed-rank. For differences between groups, we used the Mann-Whitney test. Given the normal distribution in immunohistochemistry data, the Unpaired *t*-test was used here. The correlation between expression of E-cadherin and FAM13A was tested using the Pearson’s correlation test. A *p* < 0.05 was considered significant, tested 2-sided.

## Results

### FAM13A Expression in COPD and Control Lung Tissue and Airway Epithelial Cells (AECs)

**FIGURE 1:**
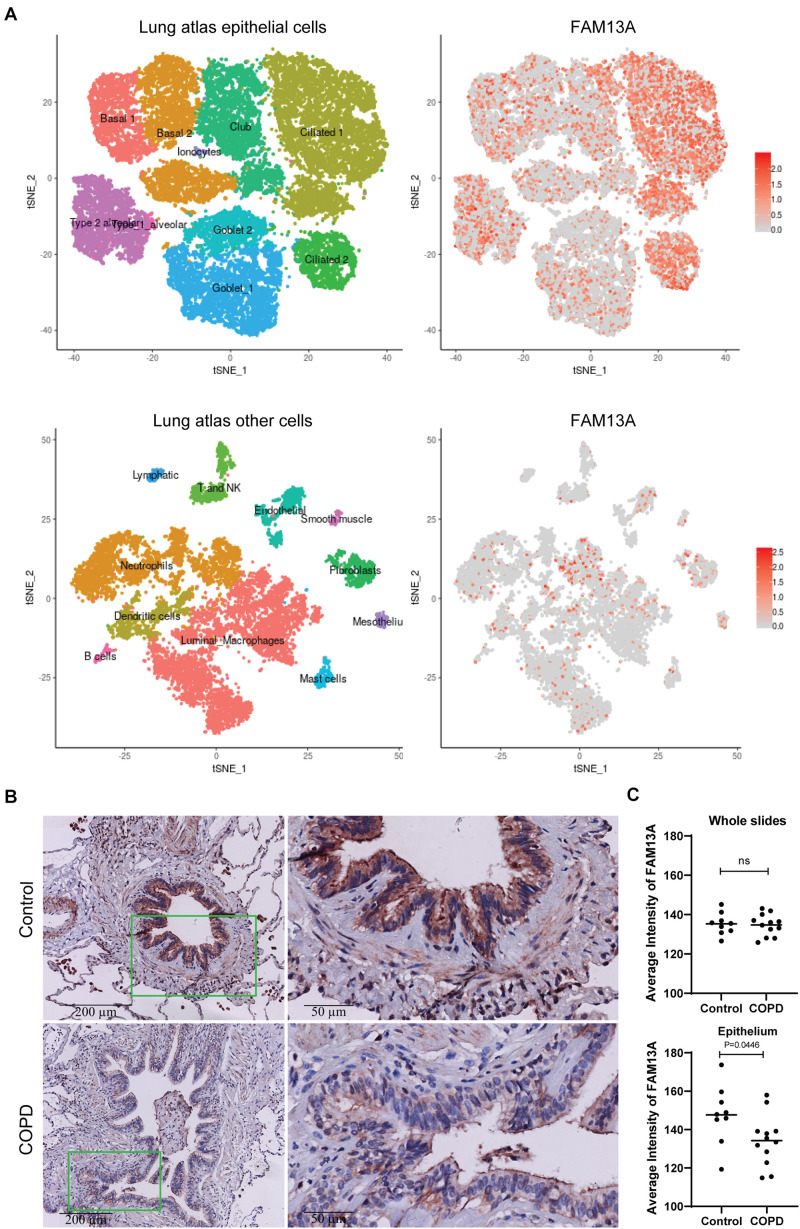
FAM13A protein expression in human lung tissue. **(A)** Expression of FAM13A in single cell RNA sequencing data of airway epithelial cells. The figure in the upper left corner shows the different clusters of airway epithelial cells. The red dots on the right figures indicate the expression of FAM13A in the different clusters of the lung cells. Figures in the lower panel show the non-epithelial cell clusters and the FAM13A expression among these clusters. **(B)** FAM13A expression in whole lung tissue and in airway epithelial cells was assessed in 9 non-COPD controls (top) and 12 COPD patients (bottom) by immunohistochemistry (IHC). Representative images are shown. **(C)** Intensity of FAM13A in the whole slide and in the epithelium of every airway in the tissue. The average intensity of all airways per donor is presented. Each dot represents one subject. Data are shown as mean. Significant differences were assessed by Unpaired *t*-test and *p*-value is indicated.

We first assessed which lung cell types express FAM13A using an online data tool, the human lung atlas containing single cell sequencing data (Vieira Braga et al., 2019). We observed strong expression of FAM13A in the airway epithelium, mainly ciliated cells. Additionally, fibroblasts and neutrophil subsets substantially expressed FAM13A (Figure 1A). Next, we assessed protein expression of FAM13A in lung tissue of COPD patients and non-COPD control by immunohistochemistry. In line with the gene expression, FAM13A protein was strongly expressed in the airway epithelium, and also detected in infiltrated immune cells and stromal cells, including smooth muscle (Figure 1B). The intensity of FAM13A expression was not different when analyzing whole lung tissue, but importantly, it was significantly lower in airway epithelium of COPD patients compared to in controls (Figure 1C).

**FIGURE 2:**
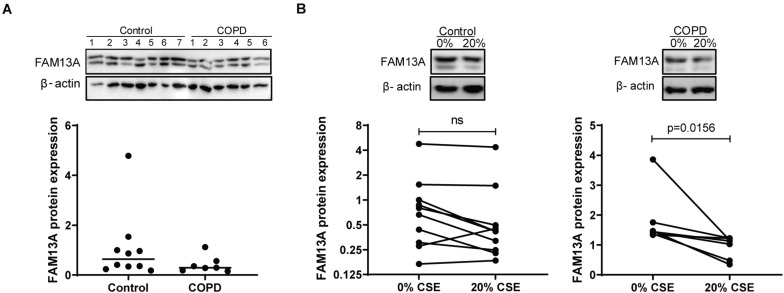
FAM13A expression in primary human airway epithelial cells. **(A)** Control and COPD-derived AECs were seeded in duplicate, grown to confluence, and incubated with hormone-free media for 24 h. FAM13A levels were detected by western blot in total cell lysates, quantified by densitometry and normalized to β-actin as the loading control. Medians are indicated, control *n* = 10, COPD *n* = 7. A representative blot is shown. Significant differences were assessed by the Mann-Whitney test. **(B)** Control and COPD-derived AECs were cultured and hormonally deprived as described above, and incubated with or without 20% cigarette smoke extract (CSE) for 24 h. FAM13A levels were detected and quantified as for panel A. Each dot represents one subject, control *n* = 10, COPD *n* = 7. Representative blots are shown. Significant differences were assessed by the Wilcoxon signed rank test and *p*-value is indicated.

In cultured AECs from COPD patients and non-COPD controls, FAM13A protein levels were not significantly different between COPD patients and non-COPD controls (Figure 2A) at baseline. Next, we exposed the cells to CSE in a concentration of 20%, as was previously used to induce pro-inflammatory responses without causing cell death (Di Vincenzo et al., 2018). Here, FAM13A levels were significantly decreased upon 24-h exposure to 20% CSE in COPD-derived primary human AECs, but not in control-derived AECs (Figure 2B).

### Expression of FAM13A in Human Bronchial Epithelial 16HBE14o-Cells

**FIGURE 3:**
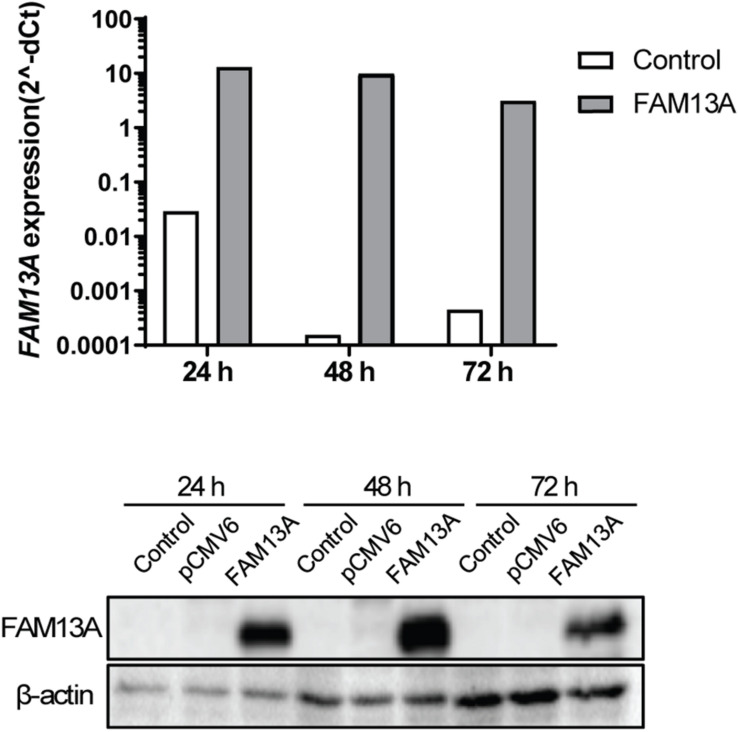
Overexpression of FAM13A in 16HBE14o- cells. 16HBE14o- cells were seeded in duplicate, transfected with or without FAM13A overexpression plasmid and harvested at 24, 48, and 72 h after the transfection. FAM13A overexpression was confirmed at mRNA level (top) and protein level (bottom). FAM13A mRNA expression levels were measured by qPCR, corrected for the housekeeping genes B2M and PPIA and normalized to control (2^− ΔCt^). FAM13A protein expression levels were measured using western blot. β-actin was used as the loading control. A representative blot is shown.

To study the functional role of FAM13A in airway epithelial cells, we overexpressed FAM13A in 16HBE14o- cells. We have previously validated the use of this cell line as model for primary AECs (Heijink et al., 2010a, 2013). FAM13A was overexpressed upon transfection, as confirmed by the increased FAM13A expression at mRNA and protein level already after 24 h (Figure 3). Overexpression of FAM13A was not affected by exposure to 10% CSE (Supplementary Figure 1).

### Increased Electrical Resistance upon FAM13A Overexpression in 16HBE14o-Cells

**FIGURE 4:**
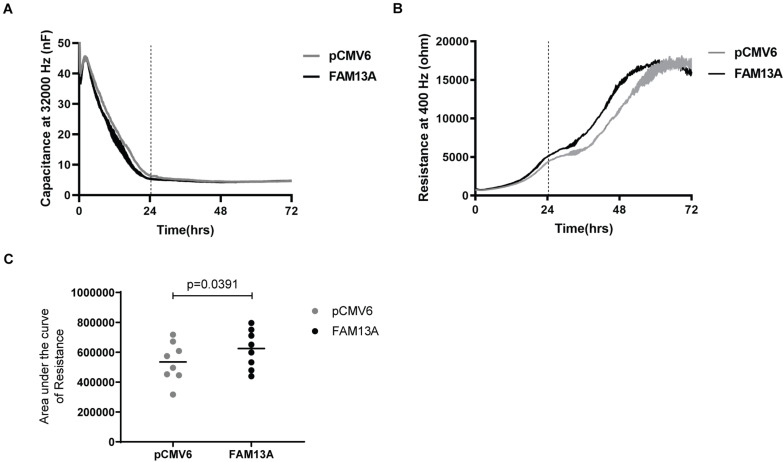
Significantly more rapid building-up of electrical resistance upon FAM13A overexpression in 16HBE14o- cells. 16HBE14o- cells were transfected with FAM13A overexpression plasmid or empty vector (pCMV6). After 24 h, the cells were trypsinized and seeded in duplicates into ECIS arrays. The high-frequency capacitance and low-frequency resistance were monitored over 72 h after seeding. **(A)** High-frequency capacitance and **(B)** low-frequency resistance values of a representative ECIS experiment. The vertical line indicates the time when cells reached confluence as indicated by the stabilization of high-frequency capacitance. Data are shown as mean ± SEM of two duplicates in the ECIS array. **(C)** The area under the curve (AUC) of resistance values from 0 to 72 h was calculated and depicted as mean and individual experiments (*n* = 8). Significant differences were assessed by the Wilcoxon signed rank test and *p*-value is indicated.

To investigate if altered FAM13A expression may contribute to abnormalities observed in airway epithelial integrity in COPD, we studied whether FAM13A overexpression alters epithelial barrier function by monitoring the formation of cell-cell contacts in 16HBE14o- cells using ECIS. As observed by the stabilization of capacitance, cells reached confluence at ∼24 h after seeding (Figure 4A). After 24 h, we observed a strong increase in resistance, which reached a plateau at ∼72 h (Figure 4B). In cells overexpressing FAM13A, resistance increased more rapidly compared to empty vector-transfected cells as reflected by the significant increase in area under the curve (Figure 4C).

### Increased Junctional Protein Levels Upon Overexpression of FAM13A in 16HBE14o-Cells

**FIGURE 5:**
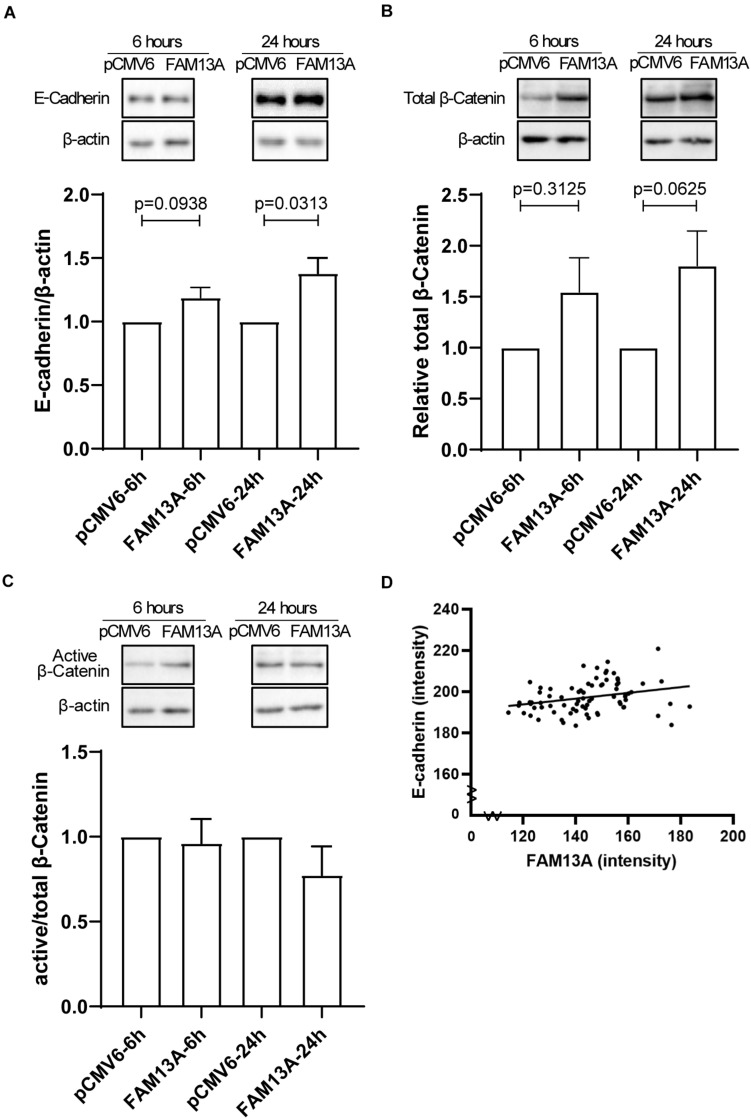
Expression of adherens junction protein E-cadherin is increased upon overexpression of FAM13A in 16HBE14o- cells and correlates with FAM13A in human lung tissue. 16HBE14o- cells were seeded in duplicate, transfected with FAM13A overexpression plasmid or empty vector (pCMV6) and harvested at 6 and 24 h after transfection. **(A)** E-cadherin protein expression levels were measured using western blot, quantified by densitometry and normalized to β-actin as loading control (mean ± SEM, *n* = 6). Total β-catenin **(B)** and active/non-phospho-β-catenin expression levels were assessed by western blot, quantified by densitometry and normalized to β-actin as loading control. **(C)** The ratio of active/non-phospho-β-catenin to total β-catenin is depicted. Representative blots are shown. Significant differences were assessed by the Wilcoxon signed rank test and the *p*-value between significant different conditions is as indicated. **(D)** The expression of E-cadherin in human lung tissue was assessed by immunohistochemistry comparable to FAM13A as described above. The correlation between the expression of FAM13A and E-cadherin in the same airway was tested using the Pearson’s correlation test. Each dot represents one airway. Number of data pairs = 77, *r* = 0.2812, *p* = 0.0132.

E-cadherin-mediated junctions are crucial for epithelial barrier formation (Nawijn et al., 2011) and are stabilized by β-catenin. We found that FAM13A overexpression in 16HBE14o- cells significantly increased the expression of E-cadherin at 24 h (Figure 5A). We observed a similar trend for total β-catenin (Figure 5B), while the expression of active β-catenin was not affected as indicated by active/non-phospho β-catenin levels (Figure 5C). Lower expression of E-cadherin has previously been observed in COPD (Oldenburger et al., 2014). Interestingly, we found a positive correlation between expression of FAM13A and E-cadherin in lung tissue, which supports a supposed role of FAM13A in the regulation of E-cadherin expression (Figure 5D).

### Cigarette Smoke-Induced CXCL8 Secretion Upon FAM13A Overexpression in 16HBE14o-Cells

**FIGURE 6:**
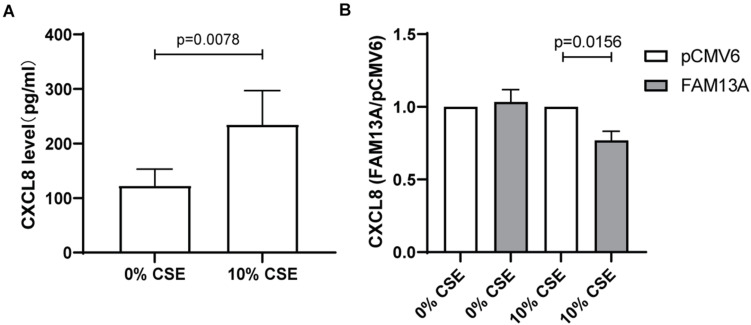
Suppressive effect of FAM13A on CSE-induced CXCL8 secretion in 16HBE14o- cells. 16HBE14o- cells were seeded in duplicate, transfected with FAM13A overexpression plasmid or empty vector (pCMV6), serum deprived overnight and exposed to 10% CSE for 24 h. CXCL8 secretion levels were measured using ELISA. **(A)** Absolute CXCL8 levels in pCMV6 transfected cells upon CSE exposure. **(B)** CXCL8 secretion levels of FAM13A overexpressing cells were measured as above and normalized to the levels of pCMV6 cells of each CSE concentration, respectively, (*n* = 8). Data are shown as mean ± SEM (*n* = 8). Significant differences were assessed by the Wilcoxon signed rank test and the *p*-value is indicated.

Finally, we investigated whether overexpression of FAM13A is able to reduce cigarette smoke-induced CXCL8 secretion. We used cigarette smoke in a concentration of 10%, as the higher concentration of 20% was shown to induce cell death in the 16HBE14o- cell line (Supplementary Figure 2). We observed that exposure to 10% CSE induced a significant increase in CXCL8 secretion in cells transfected with empty vector control pCMV6 (Figure 6A). Upon overexpression of FAM13A, the baseline secretion of CXCL8 was not significantly affected, but the CSE-induced CXCL8 release was significantly reduced compared to pCMV6 (Figure 6B).

## Discussion

In this study, we investigated the functional role of the COPD susceptibility gene FAM13A in airway epithelial barrier integrity and pro-inflammatory responses. Our data show that the intensity of FAM13A protein expression is significantly lower in the airway epithelium of COPD patients compared to non-COPD controls. In cultured airway epithelial cells, FAM13A protein expression was downregulated upon CSE exposure in COPD-derived but not in control-derived cultures. Overexpression of FAM13A led to more rapid constitution of cell-cell contacts, which may be the consequence of increased junctional E-cadherin expression. In addition, overexpression of FAM13A reduced CSE-induced CXCL8 secretion, suggesting an anti-inflammatory role. Overall, our results suggest a protective role of FAM13A in the airway epithelium with respect to the features of COPD and cigarette smoke leads to lower FAM13A levels in COPD.

FAM13A was identified in multiple GWA studies on lung function and COPD (Hobbs et al., 2017). Our group previously identified a single nucleotide polymorphism (SNP) located in gene FAM13A to be negatively associated with lung function in never-smokers. Additional, eQTL analysis showed that this SNP is associated with higher gene expression of FAM13A in human lung tissue (van der Plaat et al., 2017). These previous findings suggest higher levels of FAM13A in COPD lungs, which is partly in line with the findings of Jiang et al. (2016) who found higher FAM13A protein, but not mRNA levels in lung tissue of COPD patients compared to healthy ex-smokers. They suggest a detrimental instead of a protective role of FAM13A in the onset or severity of COPD, which is in contrast to our findings. However, their observations were done by western blotting in whole lung tissue, where FAM13A is also expressed in other cell types than the airway epithelium. For instance, an increase in smooth muscle mass could result in higher FAM13A expression in stromal cells, accounting for the differences observed between COPD and control. When assessing FAM13A in whole lung tissue, we did not observe a significant difference between COPD patients and controls. However, when specifically assessing the expression of FAM13A in the airway epithelium, we observed lower expression in COPD patients compared to non-COPD controls. In line, in airway epithelial cultures from COPD patients, but not in controls, we observed that smoke exposure significantly decreased FAM13A expression. Potentially, there is an interaction between the FAM13A gene and exposure to cigarette smoke, resulting in differential expression upon smoking, especially in epithelial cells which first encounter cigarette smoke. However, further studies are required to study this potential interaction in bronchial epithelium of smoking individuals. Together with the lower expression of FAM13A in COPD airway epithelium, our data suggest that cigarette smoking results in lower FAM13A expression in airway epithelial cells in COPD, subsequently leading to impaired epithelial barrier recovery and increased CXCL8 release, a key attractant of neutrophils.

Our findings that FAM13A advanced the formation of the epithelial barrier may reflect improved recovery of cell-cell contacts, supporting reinforcement of the epithelial barrier upon environmental insults. These effects may be explained by effects on β-catenin stability associated with higher E-cadherin expression. β-catenin is localized at the cytoplasmic side of adherens junctions and stabilizes E-cadherin (Hartsock and Nelson, 2008; Heijink et al., 2010b), while E-cadherin mediated cell-cell contacts are crucial for the formation of other epithelial junctions. Upon loss of E-cadherin, β-catenin is liberated from adherens junctions and translocated to the nucleus, activating transcription of various genes, including CXCL8 (Lévy et al., 2002; Heijink et al., 2014), but only when its phosphorylation and subsequent degradation is prevented. However, active/non-phosphorylated levels of β-catenin were not affected by FAM13A overexpression, which may indicate that β-catenin was mainly localized at epithelial junctions. It has been shown that both adherens and tight junctions are disrupted in airway epithelial cells from severe COPD patients compared to control subjects (Heijink et al., 2014; Oldenburger et al., 2014), suggesting an impaired epithelial barrier function in COPD patients. This may be due to an intrinsic deficiency to reconstitute cell-cell contacts upon epithelial damage, and lower expression of FAM13A may contribute to impaired recovery of cell-cell contacts. We speculate that FAM13A increased junctional-localized β-catenin and the expression of E-cadherin, which subsequently benefits the formation of adherens junctions and may thus advance the formation and/or recovery of the epithelial barrier. Such a role was supported by the positive correlation between FAM13A and E-cadherin expression in the airway epithelium in lung tissue.

Loss of epithelial barrier function may increase cell vulnerability to inhaled toxins such as cigarette smoke and lead to cellular damage, increased oxidative stress and subsequent release of neutrophil attractant CXCL8 (Heijink et al., 2013). Given the involvement of FAM13A in the regulation of oxidative stress and mitochondrial function (Hawkins and Mora, 2017; Jiang et al., 2017), we assessed the effects of FAM13A overexpression on CXCL8 release. We observed that FAM13A overexpression reduced the CSE-induced increase of CXCL8 levels in 16HBE14o- cells. Thus, FAM13A may suppress pro-inflammatory responses to cigarette smoking in airway epithelium and subsequently reduce neutrophilic infiltration.

A limitation of our study is that the functional studies were performed in the cell line 16HBE14o-, because of the limited availability of primary cells. However, we have previously shown that this cell line models the pro-inflammatory response of primary cells accurately (Heijink et al., 2013). Furthermore, 16HBE14o- cells form strong cell-cell contacts, which makes them highly suitable to study epithelial barrier function (Heijink et al., 2010a). In addition, 16HBE14o- express low endogenous levels of FAM13A protein, which makes them suitable for overexpression studies. Another limitation of the current study is the use of submerged epithelial cultures to assess effects of cigarette smoke extract on FAM13A expression instead of using air-liquid interface cultures exposed to gaseous-phase cigarette smoke. It is difficult to assess the most relevant method of cigarette smoke exposure. Nevertheless, previous studies from our group have shown that lipid-soluble compounds present in CSE, which we speculate to potentially represent components present in epithelial lining fluid, include aldehydes, one of the most toxic compounds of CS. These lipid-soluble components were shown to be responsible for the generation of reactive oxygen species within the epithelial cells and induction of mitochondrial damage (Hoshino et al., 2001; van der Toorn et al., 2009).

In conclusion, we found lower expression of FAM13A in airway epithelium of COPD patients. This may contribute to the pathogenesis of the disease, as FAM13A was shown to have a protective role in human airway epithelium, advancing the formation of intercellular contacts and suppressing epithelial pro-inflammatory responses upon cigarette smoke exposure. This study sheds new light on the function of FAM13A in the development of COPD and translates genetic associations to potential biological function, identifying FAM13A as a potential target for intervention.

## Data Availability Statement

The raw data supporting the conclusions of this article will be made available by the authors, without undue reservation.

## Ethics Statement

Ethical review and approval was not required for the study on human participants in accordance with the local legislation and institutional requirements. Written informed consent for participation was not required for this study in accordance with the national legislation and the institutional requirements.

## Author Contributions

QC, MV, HB, and IH contributed to the conception and design of the study. QC, MV, KON, and JN performed the experiments. C-AB supplied the lung tissue, and involved in formatting the protocol of staining and image analysis. QC, MV, and IH formatted analysis and interpretation and wrote the first draft of the manuscript. All authors contributed to manuscript revision, read, and approved the submitted version.

## Conflict of Interest

The authors declare that the research was conducted in the absence of any commercial or financial relationships that could be construed as a potential conflict of interest.

## Footnotes

1 https://www.umcg.nl/SiteCollectionDocuments/English/Researchcode/umcg-research-code-2018-en.pdf

2 www.federa.org

3 http://rsb.info.nih.gov/ij/download.html
