# Supplementary material for: A Protective Role of FAM13A in Human Airway Epithelial Cells Upon Exposure to Cigarette Smoke Extract
Source: Front Physiol. 2021 Jun 7;12:690936. doi: 10.3389/fphys.2021.690936 (PMC8215130; doi:10.3389/fphys.2021.690936)
Supplement: Supplementary file 1 [file Data_Sheet_1.PDF]

A Protective Role of FAM13A in Human Airway Epithelial Cells Upon Exposure to Cigarette Smoke Extract

Qing Chen[1,2]*, Maaike de Vries[2,3], Kingsley Okechukwu Nwozor[1,2],

Jacobien A.Noordhoek[1,2,4], Corry-Anke Brandsma[1,2],

H. Marike Boezen[2,3], Irene H. Heijink[1,2,4]

Online data supplement

MATERIALS AND METHODS

Annexin V/ PI staining

To assess the cell viability after transfection and CSE exposure, 16HBE14o- cells were stained for annexinV–FITC and propidium iodide (PI) according to the manufacturer’s instructions (IQ products, Groningen, The Netherlands) and analyzed by flow cytometry (BD FASCCalibur, BD Netherlands). In short, 16HBE14o- cells were harvested by trypsinizing after CSE exposure, washed twice with Washing Buffer (420201, BioLegend, Netherlands) and resuspended in 150 µL Annexin-V-Binding Buffer (422201, BioLegend, Netherlands). AnnexinV–FITC and /propidium iodide (PI) were added to the cell suspension and measured by flow cytometry.

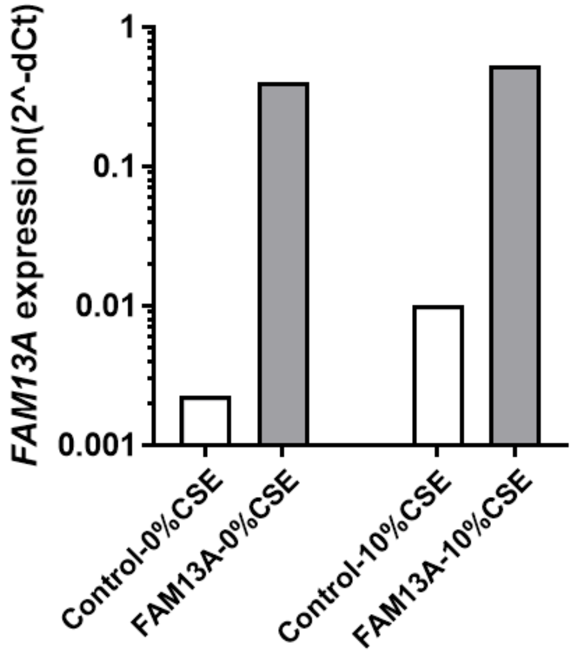

Figure S1. *FAM13A* expression in 16HBE14o- cells at baseline and after CSE exposure. 16HBE14o- cells were seeded at a density of 1 × 10^5 per well in duplicates in 24-well plates, transfected with *FAM13A* overexpression plasmid or empty vector control (pCMV6) 24 hours after seeding, serum deprived 24 hours after transfection, and exposed to 0% or 10 % CSE after 12 hours of serum deprivation. After 24 hours of CSE exposure, the cells were harvested for *FAM13A* assessment using qPCR. *FAM13A* levels were related to the housekeeping genes *B2M* and *PPIA* and expressed as $2^{-deltaCt}$, n=1.

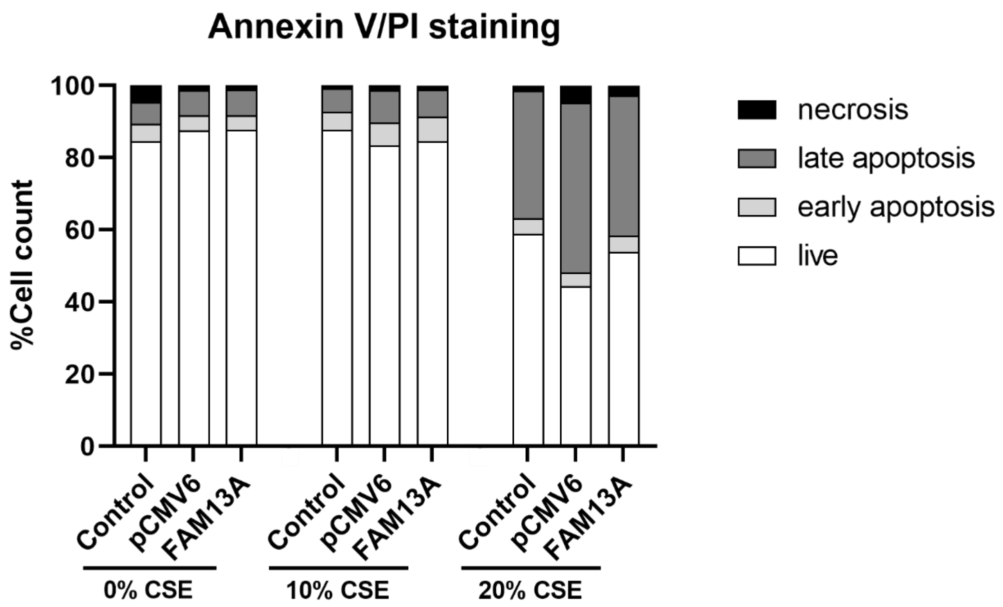

Figure S2. Viability of 16HBE14o- cells upon exposure to cigarette smoke extract (CSE). 16HBE14o- cells were seeded at a density of 1 × 10^5 per well in duplicates in 24-well plates, transfected with *FAM13A* overexpression plasmid or empty vector control (pCMV6) 24 hours after seeding, serum deprived 24 hours after transfection, and exposed to 0% or 10 % CSE after 12 hours of serum deprivation. After 24 hours of CSE exposure, the cells were stained with Annexin V/PI and measured by flow cytometry. Necrotic cells: Annexin FITC-, PI+; Late apoptosic cells: Annexin FITC+, PI+; Early apoptotic cells: Annexin FITC+, PI-; Live cells/ viable cells: Annexin FITC-, PI-. Data shown as mean, n=4.
